# A study on the epidemiology of brucellosis in bovine population of peri-urban and rural areas of district Multan, southern Punjab, Pakistan

**DOI:** 10.1186/s12917-024-03880-9

**Published:** 2024-01-31

**Authors:** Mian Muhammad Awais, Gohar Khadim, Masood Akhtar, Muhammad Irfan Anwar, Abdul Sammad Ali Khan Shirwany, Ahmad Raza, Abdul Razzaq, Zahida Fatima, Muhammad Amjad Ali, Muhammad Sibtain Bhatti

**Affiliations:** 1 One Health Research Laboratory, Department of Pathobiology, Faculty of Veterinary Sciences, Bahauddin Zakariya University, Multan, Pakistan; 2 Animal Sciences Division, Pakistan Agricultural Research Council, Islamabad, Pakistan; 3 Department of Clinical Sciences, Faculty of Veterinary Sciences, Bahauddin Zakariya University, Multan, Pakistan; 4 Livestock and Dairy Development Department, Directorate of Multan Division, Multan, Pakistan

**Keywords:** Brucellosis; prevalence, Risk determinants, i-ELISA, Molecular detection, Multan-Pakistan

## Abstract

**Background:**

Brucellosis is a zoonotic disease caused by a bacterial pathogen belonging to the genus *Brucella*. It is one of the most frequent bacterial zoonoses globally but unfortunately, it is still considered as a neglected disease in the developing world. Keeping in view, this study was conducted to determine the prevalence and risk determinants of brucellosis in large ruminants of peri-urban and rural areas of district Multan-Pakistan. For this purpose, blood samples (*n* = 490) were collected from the cattle (*n* = 245) and buffalo (*n* = 245) population of the study area and subjected to preliminary screening of brucellosis using local and imported RBPT reagents. All the samples were further analyzed using commercially available multi-specie indirect ELISA kit followed by their confirmation by PCR using genus and species-specific primers. Data obtained from lab analysis and questionnaires were subjected to statistical analysis for Pearson Chi-square, Odds Ratio and Confidence intervals (95%).

**Results:**

The results showed that the maximum seropositivity was recorded with local RBPT reagent (VRI, Pakistan; 12.45%; 95%CI = 9.72–15.65%) followed by RBPT-IDEXX (12.24%; 95%CI = 9.52–15.45%) and RBPT-ID.vet (11.84%; 95%CI = 9.18–14.95%) however statistical difference was non-significant (*P* = 0.956). The ELISA results showed an overall seroprevalence rate of 11.22% (95%CI = 8.59–14.33%) with comparatively higher rate in cattle (12.65%; 95%CI = 8.82–17.44%) as compared to buffaloes (9.80%; 95%CI = 6.49–14.15%). The PCR analysis confirmed the presence of genus *Brucella* in all seropositive samples whereas frequency of *B. abortus* and *B. melitensis* in seropositive samples was 80% and 20%, respectively. The co-existence of both species was also observed in 5.45% samples. The statistical analysis showed a significant association of bovine brucellosis with herd size, breed, reproductive disorders, mode of insemination, educational status and farmers’ awareness about brucellosis (*P* < 0.05). Conversely, locality, age, weight, gender, pregnancy status, parity and puberty status had no associations with brucellosis (*P* > 0.05).

**Conclusion:**

In conclusion, brucellosis is prevalent in large ruminants of district Multan, Pakistan. It is suggested to devise and implement stringent policies for the effective control and prevention of brucellosis in the region. Further, the current situation also warrants the need to strengthen interdisciplinary coordination among veterinarians and physicians in one health perspective to ensure and strengthen the human and animal health care systems in the region.

## Background

Brucellosis is an important bacterial disease which affects domesticated animals including ruminants, swine and dogs with zoonotic implications [1, 2]. It is caused by different species of genus *Brucella* (*B*.) from which *B*. *abortus*, *B*. *melitensis, B. canis* and *B*. *suis* are the most important species with respect to their zoonotic potential [3]. It has worldwide geographical distribution but more predominant in developing countries [4]. Approximately, 0.5 million people are infected by this disease annually [5]. In human beings, it is mainly transmitted by close contact with affected animals, their secretions, consumption of contaminated meat, milk and other dairy products whereas in animals, it is transmitted by direct or indirect contact of healthy and infected animals with each other causing abortion and infertility in their primary natural hosts [6, 7]. It has also been established as an occupational health hazard for livestock farmers and animal healthcare professionals [8]. In endemic regions of the world, it causes significant economic losses in livestock sector in terms of poor production performance, infertility, and abortion in affected animals. Additionally, it also exerts a negative impact on socio-economic development of affected countries by affecting the human health care systems, restrictions on trade of animals/their products and tourism industry [1]. The low- and middle-income countries of Asia (including Pakistan), the Middle East, the Mediterranean rim (Spain, Greece, and Portugal), Central and South America and Africa are reportedly endemic for brucellosis, but some developed countries of the world have also been succeeded in preventing or eradication of this zoonotic disease by taking strict control measures [9, 10].

In the livestock industry, management, environmental and animal factors contribute to the occurrence of this disease. The management factors may include vaccination, animal handling, farm hygiene, screening of newly purchased animals, breeding practices, milking strategies, size of the herd and production systems [8, 11]. On the other hand, animal factors include sex, breed, age and abortion history whereas environmental factor mainly comprise the agro-ecological location of animals [12, 13]. Literature also revealed that occurrence of this disease is high in pastoral grazing regions as compared to urban areas [14]. Moreover, survival potential of *Brucella* in cold and humid environments is another important factor for the transmission of this disease in livestock and humans [15]. The human behavioral factors such as unhygienic conditions and other activities that cause the exposure of people to infected animals and their products enhances the chances of disease in humans [16]. On the whole, the farmers, abattoir, laboratory workers, and people that are involved in the livestock industry may be regarded as high risk populations [17]. The other risk factors which serve as main barriers in controlling brucellosis are conventional livestock policies, political influences, inadequate implementation of control measures, usage of old traditional practices, consumption of unpasteurized dairy products, changes in the animal husbandry system and insufficient measures for reporting, diagnosis and surveillance of this disease [10]. The appropriate treatment and prevention of this disease are indispensable to avoid economic losses in the livestock sector. The developing countries are lacking behind in implementing the eradication strategies against brucellosis [18–20]. Although the World Health Organization (WHO) and World Organization for Animal Health (WOAH) have devised recommendations for the eradication of brucellosis, but it is still considered a serious health threat in developing/underdeveloped countries because control measures for the eradication of brucellosis are expensive, laborious, and time-consuming. However, in endemic countries, its occurrence can be avoided by vaccination and slaughtering/culling strategies [21].

Due to limited diagnostic facilities, brucellosis is still considered a neglected disease in most parts of the world including Pakistan [22, 23]. Due to this reason, the dynamics and zoonotic importance of disease are not fully explored to formulate any stringent strategy to overcome this important health issue. Under prevailing conditions, field veterinarians rely on Rose Bengal Plate Test (RBPT) and Milk Ring Test (MRT) for the rapid diagnosis of brucellosis in large ruminants [24]. Anyhow, it is always recommended to use RBPT with other serological tests (with high sensitivity and specificity) for an accurate diagnosis to avoid false-positive as well as false negative results [25]. So, for effective control and prevention, it is imperative to conduct studies on brucellosis using sophisticated serological and molecular diagnostic tools to generate baseline data on its prevalence and associated risk factors. In Pakistan, some previous studies have reported the prevalence and risk determinants of brucellosis in some selected districts of the country [26–28], but information regarding the epidemiology of brucellosis is scarce/ limited in southern Punjab which is the hub of livestock and inhabits approximately more than 50% of total livestock population of the province Punjab. Keeping in view, this study was conducted to assess the prevalence and associated risk factors of brucellosis in large ruminants of district Multan, Punjab-Pakistan using sero- and molecular diagnostic techniques. It is anticipated that data generated from this study will be helpful for the livestock policy/decision-making bodies to formulate effective strategies to control brucellosis in large ruminants.

## Results

### Screening and comparison of brucellosis using different commercially available RBPT antigens

In overall ruminant population, the maximum seropositivity was recorded with RBPT-VRI (12.45%; 95%CI = 9.72–15.65%) followed by RBPT-IDEXX (12.24%; 95%CI = 9.52–15.45%) and RBPT-ID.vet (11.84%; 95%CI = 9.18–14.95%). The statistical analysis revealed that difference was non-significant (*P* = 0.956) which indicated that all the antigens are equally good for preliminary screening. A similar trend was observed in species-wise analysis (Table 1).

**Table 1:** Screening and comparison of brucellosis using different commercially available RBPT antigens

	% Seropositivity with RBPT (n/T) (95%CI)			χ^2^	*P*-Value
	VRI	IDEXX	ID.vet		
Overall	12.45 (61/490) (9.72–15.65)	12.24 (60/490) (9.52–15.45)	11.84 (58/490) (9.18–14.95)	0.089	0.956
Cattle	15.10 (37/245) (10.86–20.10)	15.92 (39/245) (11.70-21.03)	15.51 (38/245) (11.28–20.51)	0.062	0.969
Buffalo	9.79 (24/245) (6.49–14.15)	8.57 (21/245) (5.56–12.72)	8.16 (20/245) (5.14–12.31)	0.439	0.803

RBPT = Rose Bengal Plate Test; 95%CI = 95% confidence interval; VRI = Veterinary Research Institute; χ^2^ = Chi-square

### Overall and species-wise prevalence of brucellosis

Results of analysis using multi-species Indirect ELISA kit showed that overall prevalence of brucellosis in large ruminants was 11.22% (*n* = 55/490; 95%CI = 8.59–14.33%). In species-wise analysis, it was revealed that seroprevalence of brucellosis was comparatively higher in cattle (12.65%; 95%CI = 8.82–17.44%) as compared to buffalo (9.80%; 95%CI = 6.49–14.15%) population however difference was non-significant (*P* = 0.316) (Table 2).

**Table 2:** Overall and species-wise prevalence of brucellosis using multi-species i-ELISA Kit

Large Ruminant	Total samples (n)	Positive samples (n)	% Seroprevalence (95% CI)	*P*-Value	χ^2^	OR	OR (95% CI)	
							Lower Limit	Upper Limit
Overall	490	55	11.22 (8.59–14.33)	-	-	-		
Cattle	245	31	12.65 (8.82–17.44)	0.316	1.004	1.33	0.76	2.37
Buffalo	245	24	9.80 (6.49–14.15)					

iELISA- Indirect Enzyme linked immunosorbent assay; 95%CI = 95% confidence interval; χ^2^ = Chi square; OR = Odds Ratio

### Molecular detection of Brucella species in ELISA positive samples

The ELISA positive samples were subjected to PCR for confirmation of brucellosis and detection of *Brucella* species in positive samples. The results confirmed the presence of *Brucella* in all the ELISA positive samples using genus specific primers. On the other hand, the results of PCR using species-specific primers revealed that out of total positive samples, *B. abortus* was detected in 80% (*n* = 44/55) large ruminants with overall prevalence of 8.98% (*n* = 44/490). The *B. melitensis* was detected in 20% (*n* = 11/55) of positive samples with overall prevalence of 2.24% (*n* = 11/490) in large ruminants. The co-prevalence of *B. abortus* and *B. melitensis* in positive samples was 5.45% (*n* = 3/55) with an overall prevalence of 0.61% (*n* = 3/490) in large ruminants. A similar trend was recorded in cattle and buffalo populations when analyzed separately (Table 3).

**Table 3:** Molecular detection of *Brucella* species in ELISA positive samples by Polymerase Chain Reaction

Population	Brucella Spp. % (n)	B. abortus (BA) % (n)	B. melitensis (BM) % (n)	Both BA & BM % (n)
***Out of ELISA positive samples***				
Overall	100 (55/55)	80 (44/55)	20 (11/55)	5.45 (3/55)
Cattle	100 (31/31)	80.64 (25/31)	19.36 (06/31)	3.22 (01/31)
Buffalo	100 (24/24)	79.17 (19/24)	20.83 (5/24)	8.33 (02/24)
***Out of total population***				
Overall	11.22 (55/490)	8.98 (44/490)	2.24 (11/490)	0.61 (03/490)
Cattle	12.65 (31/245)	10.2 (25/245)	2.45 (06/245)	0.41 (01/245)
Buffalo	9.80 (24/245)	7.76 (19/245)	2.04 (05/245)	0.82 (02/245)

### Farm/herd level and tehsil wise prevalence

In this study, a total of 67 farms located in peri-urban and rural areas of different tehsils of district Multan were sampled. While calculating farm/herd level prevalence, a herd/farm having at least one positive animal for *Brucella* antibodies was considered positive. Out of total, animals at 22 farms were positive for brucellosis indicating its farm level prevalence of 32.83% (95%CI = 22.00-44.82%). In tehsil wise data analysis, it was revealed that farm level prevalence was highest in Tehsil Multan (43.75%; 95%CI = 20.11–69.96%) followed by those of Shujabad (31.03%; 95%CI = 16.38-50.00%) and Jalalpur Pirwala (27.27; 95%CI = 12.60–50.00%) however the difference was non-significant (*P* = 0.545). In tehsil-wise analysis of prevalence of brucellosis, the highest prevalence rate 12.07% (95%CI = 7.85–17.66%) was recorded in large ruminants of tehsil Multan followed by those of Shujabad (11.11%; 95%CI = 7.04–16.78%) and Jalalpur Pirwala (10.34%; 95%CI = 5.91–16.35%) however the difference was non-significant (*P* = 0.887). A similar trend was recorded in cattle and buffalo populations of different tehsils, when analyzed separately (Table 4).

**Table 4:** Farm/herd level and Tehsil wise prevalence of brucellosis in large ruminants

Large Ruminants	% Prevalence (n/T) (95% CI)	*P*-Value	χ^2^	OR	OR (95% CI)	
					Lower limit	Upper limit
**Herd-Level**						
Overall	32.83 (22/67) (22.00-44.82)					
Multan	43.75 (7/16) (20.11–69.96)	0.545	1.216	2.03	0.43	10.04
Shujabad	31.03 (9/29) (16.38-50.00)			1.2	0.3	5.02
Jalalpur Pirwala	27.27 (6/22) (12.60–50.00)			1		
**Tehsil-wise**						
**Overall large ruminants**						
Multan	12.07 (21/174) (7.85–17.66)	0.887	0.239	1.19	0.56	2.59
Shujabad	11.11 (19/171) (7.04–16.78)			1.08	0.5	2.39
Jalalpur Pirwala	10.34 (15/145) (5.91–16.35)			1		
**Cattle**						
Multan	15.05 (14/93) (8.77–23.94)	0.673	0.791	1.36	0.45	4.61
Shujabad	11.00 (11/100) (5.87–18.72)			0.95	0.3	3.33
Jalalpur Pirwala	11.54 (6/52) (5.14–22.66)			1		
**Buffalo**						
Multan	8.64 (7/81) (3.91–16.91)	0.862	0.298	0.88	0.27	2.82
Shujabad	11.27 (8/71) (5.01–20.76)			1.18	0.37	3.68
Jalalpur Pirwala	9.68 (9/93) (4.95–17.43)			1		

95%CI = 95% confidence interval; χ^2^ = Chi-square; OR = odds ratio

### Association of demographic parameters with the prevalence of brucellosis

Results showed highest prevalence of brucellosis in large ruminants aged > 8 years (15.91%; OR = 1.81; 95%CI OR = 0.62–4.62) followed by those of ≤ 4 years (13.67%; OR = 1.52; 95%CI OR = 0.77 − 0.62) and > 4but ≤ 8 years (9.45%; OR = 1) whereas the statistical difference between different age groups was non-significant (*P* = 0.249). A similar non-significant association was recorded when age-wise data was analyzed for cattle (*P* = 0.786) and buffalo (*P* = 0.132) populations, separately. The gender-wise analysis showed higher prevalence of brucellosis in females (11.30%) of large ruminants as compared to males (10.00%) however the difference was statistically non-significant (*P* = 0.826; OR = 1.10; 95%CI OR = 0.37–4.91). Similarly, a non-significant association was recorded between gender and prevalence of brucellosis in cattle (*P* = 0.572; OR = 0.61; 95%CI OR = 0.14–4.52) and buffalo (*P* = 0.489; OR = 1.80; 95%CI OR = 0.34–44.75) populations when their data was analyzed separately. In breed-wise analysis of cattle population, highest prevalence was recorded in non-descripts (20.69%; OR = 2.08; 95%CI OR = 0.61–9.12) and the lowest prevalence was recorded in Sahiwal breed (6.49%; OR = 0.56; 95%CI OR = 0.11–3.01) and the difference of prevalence between different breeds was statistically significant (*P* = 0.039). In buffalo population, non-descript buffaloes showed a higher prevalence rate (25.81%) as compared to Nili-Ravi breed (7.48%) and the difference was statistically significant **(***P* = 0.001; OR = 0.23; 95%CI OR = 0.09–0.64). Results also revealed a non-significant association (*P* > 0.05) between body weight and prevalence of brucellosis in large ruminants. Depending upon number of parities, data was divided into 2 groups of females viz. i). with ≤ 3 parities and ii). having > 3 parities. Analysis revealed non-significant association of number of parities with prevalence of brucellosis both in cattle and buffalo populations (*P* > 0.05; OR = 1.17; 95%CI OR = 0.61–2.36). The correlation analysis of brucellosis with physiological status of females revealed slightly higher prevalence of brucellosis in pregnant females (12.24%) as compared to non-pregnant females (11.19%) whereas the difference was statistically non-significant (*P* = 0.826; OR = 1.13; 95%CI OR = 0.41–2.63). The herd size was found to be significantly associated with prevalence of brucellosis in large ruminants (*P* < 0.05). Overall, the prevalence rate was highest in animals of herd size > 100 heads (OR = 1) and lowest in animals with 11–100 heads herd size (OR = 0.14; 95%CI OR = 0.06–0.29). With respect to puberty status of females, a non-significant association was recorded between puberty status and prevalence of brucellosis (*P* > 0.05; OR = 1.73; 95%CI OR = 0.80–3.49). Based upon insemination protocol, natural breeding (OR = 3.45; 95%CI OR = 1.31–11.50) was found to be significantly associated with high prevalence of brucellosis in overall large ruminants (*P* = 0.024). Similar trend was observed in cattle population (*P* = 0.012) however in case of buffaloes, difference was non-significant (*P* = 0.483). Results also showed that prevalence of brucellosis was higher in bovines reared at farms where small ruminants were also present as compared to those reared in exclusive large ruminant farming system; however, the difference was statistically non-significant (*P* = 0.095; OR = 0.62; 95%CI OR = 0.35–1.09) (Table 5).

**Table 5:** Association of demographic parameters with the prevalence of brucellosis

Demographic Characters	% Prevalence (n/T) (95% CI)	χ^2^	*P*-Value	OR	OR (95% CI)	
					Lower limit	Upper limit
**Age (years)**						
**Overall**						
≤ 4	13.67 (19/139) (8.76–20.32)	2.777	0.249	1.52	0.77	0.62
> 4 years but ≤ 8	9.45 (29/307) (6.56–13.24)			1		
More than 8	15.91 (7/44) (7.21–29.18)			1.81	0.62	4.62
**Cattle**						
≤ 4	14.44 (13/90) (8.15–2308)	0.481	0.786	1.26	0.53	2.93
> 4 years but ≤ 8	11.80 (17/144) (7.19–18.20)			1		
More than 8	9.09 (1/11) (0.46–40.11)			0.75	0.02	5.89
**Buffalo**						
≤ 4	12.24 (6/49) (5.47–24.06)	4.052	0.132	1.75	0.51	5.4
> 4 years but ≤ 8	7.36 (12/163) (4.07–12.38)			1		
More than 8	18.18 (6/33) (8.22–34.47)			2.78	0.79	2.78
**Gender**						
**Overall**						
Female	11.30 (52/460) (8.61–14.50)	0.048	0.826	1.10	0.37	4.91
Male	10.00 (3/30) (2.77–25.96)					
**Cattle**						
Female	12.39 (29/234) (8.61–17.19)	0.319	0.572	0.61	0.14	4.52
Male	18.18 (2/11) (3.32–50.01)					
**Buffalo**						
Female	10.17 (23/226) (6.69–14.70)	0.479	0.489	1.80	0.34	44.75
Male	5.26 (1/19) (0.26–25.17)					
**Breed**						
**Cattle**						
Sahiwal	6.49 (5/77) (2.59–14.50)	8.382	0.039	0.56	0.11	3.01
Friesian	8.88 (4/45) (3.08–20.57)			0.78	0.13	4.55
Crossbred	11.11 (4/36) (3.88–25.77)			1		
Non-descript	20.69 (18/87) (12.82–30.27)			2.08	0.61	9.12
**Buffalo**						
Nili Ravi	7.48 (16/214) (4.50-11.76)	10.296	0.001	0.23	0.09	0.64
Non-descript	25.81 (8/31) (12.61–43.38)					
**Herd size (No. of heads)**						
**Overall**						
Up to 10	26.22 (16/61) (15.86–38.37)	49.128	0.000	0.9	0.39	2.05
From 11 to 100	5.07 (18/355) (3.13–7.78)			0.14	0.06	0.29
> 100	28.38 (21/74) (18.51–39.75)			1		
**Cattle**						
Up to 10	30.00 (9/30) (15.81–48.29)	32.259	0.000	0.79	0.24	2.54
From 11 to 100	5.52 (10/181) (2.83–9.76)			0.11	0.04	0.31
> 100	35.29 (12/34) (19.99–53.02)			1		
**Buffalo**						
Up to 10	22.58 (7/31) (10.26–40.06)	18.361	0.000	1	0.27	3.54
From 11 to 100	4.60 (8/174) (2.04–8.71)			0.17	0.05	0.53
> 100	22.50 (9/40) (11.84–37.97)			1		
**Parity**						
**Overall**						
≤ 3	11.75 (39/332) (8.63–15.58)	0.233	0.629	1.17	0.61	2.36
> 3	10.16 (13/128) (5.52–16.57)					
**Cattle**						
≤ 3	13.89 (25/180) (9.22–19.59)	1.607	0.205	1.95	0.71	7.05
> 3	7.41 (4/54) (2.56–17.22)					
**Buffalo**						
≤ 3	9.21 (14/152) (5.36–14.92)	0.474	0.491	0.73	0.30	1.85
> 3	12.16 (9/74) (6.26–21.31)					
**Pregnancy**						
**Overall**						
Yes	12.24 (6/49) (5.47–24.06)	0.048	0.826	1.13	0.41	2.63
No	11.19 (46/411) (8.41–14.64)					
**Cattle**						
Yes	15.00 (3/20) (4.21–36.94)	0.137	0.711	1.32	0.28	4.34
No	12.15 (26/214) (8.24–17.16)					
**Buffalo**						
Yes	10.34 (3/29) (2.87–26.87)	0.001	0.974	1.06	0.23	3.42
No	10.15 (20/197) (6.40-15.08)					
**Puberty Status**						
**Overall**						
Heifers	16.67 (11/66) (9.14–27.73)	2.210	0.137	1.73	0.80	3.49
Adults	10.41 (41/394) (7.64–13.76)					
**Cattle**						
Heifers	16.67 (5/30) (6.80-34.52)	0.579	0.447	1.53	0.47	4.14
Adults	11.76 (24/204) (7.89–16.83)					
**Buffalo**						
Heifers	16.67 (6/36) (7.51–32.03)	1.973	0.160	2.06	0.68	5.50
Adults	8.95 (17/190) (5.30-13.79)					
**Mode of insemination**						
**Overall**						
Artificial Insemination	4.55 (5/110) (1.80-10.14)	7.436	0.024	1		
Natural Breeding	14.14 (41/290) (10.39–10.57)			3.45	1.31	11.50
Not Inseminated	10.00 (6/60) (4.44–20.38)			2.32	0.56	10.08
**Cattle**						
Artificial Insemination	4.44 (4/90) (1.52–10.74)	8.814	0.012	1		
Natural Breeding	18.10 (21/116) (11.78–26.1)			4.72	1.51	19.67
Not Inseminated	14.29 (4/28) (5.02–31.61)			3.53	0.61	20.50
**Buffalo**						
Artificial Insemination	5.00 (1/20) (0.25–23.88)	1.456	0.483	1		
Natural Breeding	11.49 (20/174) (7.24–17.07)			2.46	0.35	107.54
Not Inseminated	6.25 (2/32) (1.12–19.59)			1.26	0.06	78.57
**Co-raising of small ruminants**						
No	9.19 (26/283) (6.23–13.13)	2.79	0.095	0.62	0.35	1.09
Yes	14.01 (29/207) (9.73–19.44)					

### Association of reproductive disorders with prevalence of brucellosis

Results revealed that large ruminants with abortion history showed higher prevalence rate (46.15%) as compared to those with no abortion history (9.22%) and the difference was significant (*P* = 0.000; OR = 8.38; 95%CI OR = 3.55–19.56). A similar association was observed in cattle and buffalo populations when data was analyzed separately. Similarly, some other reproductive disorders like repeat breeding history (OR = 3.42; 95%CI OR = 1.58–7.03) and retention of fetal membranes (OR = 6.44; 95%CI OR = 2.98–13.60) also showed a significant association (*P* < 0.05) with prevalence of brucellosis in large ruminants of study area (Table 6).

**Table 6:** Association of reproductive disorders with prevalence of brucellosis

Reproductive disorders	%Prevalence (n/T) 95% CI	χ^2^	OR	OR 95% CI			*P*-Value
				Lower limit		Upper limit	
**Abortion History**							
**Overall**							
Yes	46.15 (12/26) (28.20-65.92)	33.380	8.38	3.55	19.56		0.000
No	9.22 (40/434) (6.71–12.26)						
**Cattle**							
Yes	47.06 (8/17) (25.29–71.79)	20.290	8.17	2.75	24.04		0.000
No	9.68 (21/217) (6.29–14.37)						
**Buffalo**							
Yes	40.00 (4/10) (15.00-71.71)	10.180	6.87	1.57	27.08		0.001
No	8.80 (19/216) (5.51–13.28)						
**Repeat Breeding History**							
**Overall**							
Yes	26.67 (12/45) (14.84–41.10)	11.741	3.42	1.58	7.03		0.001
No	9.64 (40/415) (7.01–12.81)						
**Cattle**							
Yes	28.00 (7/25) (12.76–47.94)	6.279	3.31	1.16	8.66		0.012
No	10.53 (22/209) (6.74–15.40)						
**Buffalo**							
Yes	25.00 (5/20) (10.41–47.40)	5.274	3.51	1.02	10.44		0.022
No	8.74 (18/206) (5.40-13.43)						
**History of retention of fetal membranes**							
**Overall**							
Yes	38.88 (14/36) (24.16–55.70)	29.640	6.44	2.98	13.60		0.000
No	8.96 (38/424) (6.51–12.06)						
**Cattle**							
Yes	42.85 (6/14) (20.00-68.84)	12.728	6.37	1.90	20.35		0.000
No	10.45 (23/220) (6.87–15.09)						
**Buffalo**							
Yes	36.36 (8/22) (18.70-58.24)	18.283	7.12	2.47	19.80		0.000
No	7.35 (15/204) (4.19–11.84)						

### Association of educational status and awareness level of farmers with prevalence of brucellosis

The large ruminants kept by farmers having poor educational status (≤ matriculation) showed higher prevalence rate (14.28%) as compared to those with educational status above matriculation (3.57%) and difference was statistically significant (*P* = 0.001; OR = 4.37; 95%CI OR = 1.86–12.98). Similarly, lack of awareness about brucellosis also had a significant association with prevalence of brucellosis in large ruminants (*P* = 0.013; OR = 0.35; 95%CI OR = 0.13–0.79) (Table 7).

**Table 7:** Association of educational status and awareness level of farmers with prevalence of brucellosis

Groups	Total samples (n)	Positive samples (n)	Negative samples (n)	% Prevalence (95% CI)	P-value	χ^2^	OR	OR (95% CI)	
								Lower	Upper
**Educational status of Farmers**									
≤ Matric	350	50	300	14.28 (10.90-18.45)	0.001	11.52	4.37	1.86	12.98
Above Matric	140	5	135	3.57 (1.41–7.96)					
**Awareness about Brucellosis**									
Yes	120	6	114	5.00 (2.20-10.52)	0.013	6.179	0.35	0.13	0.79
No	370	49	321	13.24 (10.03–17.09)					

## Discussion

Brucellosis is an infectious disease with serious zoonotic implications affecting both animals and humans around the globe [8]. It badly affects the livestock sector and results in enormous economic losses [29]. In addition to adverse effects in the livestock industry, brucellosis is also being highlighted as a potential occupational hazard in individuals associated with the livestock sector including farmers, milkers, shepherds, veterinary healthcare professionals, abattoir workers, etc. [30]. . In Pakistan, it is an increasingly significant health issue of veterinary and public health concerns because most of the rural population relies on the livestock sector to earn their livelihood and spend most of their time in close contact with animals to raise them. In this study, seropositivity of brucellosis was determined with different RBPT reagents and a non-significant difference was recorded in seropositivity rates with different antigens. However, locally produced RBPT-VRI was the cheapest one and it can be effectively used as a rapid and economical screening test under local conditions. The ELISA results revealed that the prevalence of bovine brucellosis in the target population was 11.22%. However, molecular characterization revealed higher prevalence of *B. abortus* in seropositive animals. Contrary to our findings, studies conducted in different parts of the world showed higher prevalence rates of bovine brucellosis like 14.3% in Jimma zone, Ethiopia [31], 16.3% in southwestern Nigeria [32], 28.9% in Rwanda [33],, 55% in 6 southern districts of Albania [34], 20.36% in India [35], 31% in Bahr el Ghazal region of South Sudan [36], and 27.07% in Goa, India [37]. However, lower prevalence rates had also been reported in some other countries such as 2.4% in Alage district of Ethiopia [38], 3.40% in Ngaoundéré, Cameroon [39], 9.7% in Nechisar National Park, Ethiopia [40], 0.4% in Sendafa, Oromia Special Zone, Ethiopia [41], 6.35% in Southern Cameron [42], and 5.10% in Indonesia [43]. Several factors including geographical conditions, sampling methods, breed of animals, herd size and different methods of diagnosis might be the possible reasons for the varied prevalence rates in different regions of the world. The herd-level prevalence of bovine brucellosis in Multan district was 32.83% with highest prevalence rate in herds of Tehsil Multan followed by Shujabad and Jalalpur Pirwala. The herds having at least one positive animal for *Brucella* antibodies were considered positive [44]. Previously, no herd level prevalence had been reported in the study area. However, Ali et al. [45]. and Khan et al. [46]. reported 18.6% and 58.7% herd-based prevalence rates of bovine brucellosis in the Potohar Plateau and Gujranwala districts of Pakistan, respectively. The difference might be attributed to the fact that availability of favorable climate to the bacteria which can sustain better in humid climatic conditions as compared to dry climate [47, 48]. The variation might also be correlated with differences in husbandry practices, diagnostic technique employed and density/size of herd in different regions. Our results showed a non-significant difference in tehsil wise prevalence of bovine brucellosis however higher relatively lower prevalence rates have been reported in Bannu (5.2%) [49] and prevalence of 6.6% in cattle and 1.6% in buffalo has been reported in Rawalpindi and Islamabad [50]. The varied prevalence rates in different parts of the world might be attributed to differences in geo-climatic conditions.

It is well-established that sexually mature animals remain at high risk to be infected with brucellosis [45]. This might be due to increased susceptibility under the influence of sex hormones and elevated levels of erythritol and fetal fluids in the placenta resulting in stimulated growth and replication of bacteria in the reproductive organs [51, 52]. However, young ones may also get infected while feeding on contaminated milk from infected dams. In our study, analysis revealed that the association of different age groups with prevalence of brucellosis in large ruminants was non-significant. Our findings are in agreement with those of Sima et al. [51] and Jamil et al. [26] but contrary to some other previous studies [27, 46, 53, 54]. The variation in findings of different studies might also be due to non-uniformity in categorization of animals in different age groups. In this study, the association of live body weight with prevalence of bovine brucellosis was also determined to be non-significant. No such correlation has been reported previously. The lower body weights might be linked with malnourished status and thus poor immunity of animals which might be one the possible reasons behind higher prevalence rate in animals of lesser body weight groups [55]. On the other hand, brucellosis had also been reported as a cause of reduced weight gain in affected animals [23]. A non-significant association of gender with prevalence of brucellosis was observed in bovine population of study area. In agreement to our findings, Sima et al. [51] and Khan et al. [46] also reported a non-significant difference in gender-wise prevalence of brucellosis in large ruminants. Conversely, a significant association had also been reported between animal sex and brucellosis with predominantly higher prevalence rates in females [53, 56]. In study area, farmers retain only female calves for milk production and to get next progeny whereas males are being sold out/slaughtered at early age, so we could collect relatively smaller number of sera samples from males as compared to females. Accordingly, a non-significant association in our study might be due to relatively smaller sample size of male animals. In our study, a significant association (*P* < 0.05) was recorded between prevalence of brucellosis and breeds of large ruminants. It was observed that native cattle (Sahiwal) and buffalo (Nili-Ravi) breeds showed lowest prevalence of brucellosis. On the other hand, non-descriptive breeds showed highest prevalence rates in both cattle and buffalo populations. Previously, Patel et al. [54], Holt et al. [8] and Sima et al. [51] also reported a positive association of animal breeds with prevalence of brucellosis in bovines. The lower prevalence rates in native breeds might be linked with their better adoptability to local geo-climatic conditions and genetic resistance to endemic diseases including brucellosis. It suggested that careful breeding policies should be devised for effective control of endemic diseases. Our results showed a positive association between prevalence of brucellosis and herd size of large ruminants. The medium sized herds having 11–100 animal bovine heads showed significantly lower prevalence rate as compared to large or small sized herds. Previously, Deka et al. [57] and Aulakh et al. [58] had also reported similar findings. Contrarily, Khan et al. [59] and Ali et al. [45] reported a non-significant association between herd size and prevalence of bovine brucellosis. In our study, higher prevalence in small sized herds might be correlated with poor husbandry practices in addition to lack of awareness of farmers about disease. On the other hand, in large sized herds, it might be due to indiscriminate replacement of herd from unknown sources without opting quarantine protocols along with inappropriate disease screening, hygiene and management [14, 59]. In this study, no association was found between prevalence of brucellosis and number of parities in large ruminants. Previously, Dinka et al. [60] and Khan et al. [59] also reported similar findings. However, a significant difference has been reported in prevalence of brucellosis in cattle with no, single or multiple parities [53].

A non-significant association of brucellosis with pregnancy status was recorded in this study. Previously, a non-significant association between pregnancy and brucellosis in cattle has been reported [53]. However, Khan et al. [50] reported pregnancy status as a potential risk determinant of bovine brucellosis in dairy cattle raised under peri-urban production system in different regions of Punjab-Pakistan. The difference in our study might be due to differences in breed, husbandry practices and geo-climatic conditions of study area. Our results aslo showed a non-significant association of puberty status of female ruminants with brucellosis. It can be hypothesized that young animals are more resistant to *Brucella* infection, predominantly due to passive immunity. It might also be due to latent infection in calves which could not result in sero-conversion or clinical symptoms till they reach age of puberty or sexual maturity. However, the exact phenomenon is complicated and still to be explored [61].

The insemination protocols always remained a matter of great concern with respect to sexually transmitted diseases. In our study area, majority of the farmers were practicing natural breeding to inseminate their female animals and results revealed significantly higher prevalence of brucellosis in females of large ruminants inseminated through natural breeding however specifically in case of buffaloes, the difference was non-significant (*P* > 0.05). Earlier, Deka et al. [57] also reported significantly lower prevalence in dairy animals for which artificial insemination protocol was adopted for breeding purpose. On the other hand, a non-significant association of insemination method with brucellosis in buffalo population has also been reported by Batistia et al. [62]and this is in accordance with our findings in buffalo population. In a previous study on Columbian herds, it has been reported that farms on natural breeding with bulls from non-certified herds showed significantly higher prevalence of bovine brucellosis as compared to those using artificial insemination [63]. In middle-low socio-economic settings, animals are mostly bred through natural breeding and there are no standard operating procedures to evaluate and certify their *Brucella*-free status. Similarly, semen collected at semen production units or affiliated farms is also not passed through stringent quality control checks. It arises the need to introduce the concept of routine screening of breeding bulls for sexually transmitted diseases followed by their certification. Additionally, strict quality control/assurance protocols should also be devised for semen used for artificial insemination to avoid transmission of STDs through AI.

In female population, association of brucellosis with reproductive disorders including history of abortion, repeat breeding and retained fetal membranes was also determined. Results revealed a significant association of brucellosis with all these reproductive disorders (*P* < 0.05) in both cattle and buffalo populations. Like our findings, various previous studies had also reported a significant association of reproductive disorders with prevalence of brucellosis in bovines [27, 46, 53]. Contrarily, Patel et al. [54]. reported a non-significant correlation of repeat breeding and Khan et al. [48]. reported a non-significant correlation of retained fetal membranes with brucellosis in bovines. As these reproductive problems are cardinal signs of brucellosis, and therefore these results are not surprising. However, in our study area, high prevalence might be correlated with behavior of farmers for not culling the infected animals, introduction of new animals in existing stock without screening and quarantine, higher inclination towards natural breeding and lack of awareness about disease. Due to the reason, there is an increasing threat for transmission of infection in healthy populations through contaminated environment by the excretion of *Brucella* pathogen with the aborted fetuses, reproductive fluids and retained fetal membranes of infected animals. This is also an alarming situation anticipating the zoonotic implications of brucellosis as an occupational health hazard for personals dealing with animals. The educational status and their level of awareness about bovine brucellosis were found significantly associated with prevalence of brucellosis in large ruminants. The animals raised by farmers with poor educational status and lack of awareness about disease had significantly higher (*P* < 0.05) prevalence rates of brucellosis. Similarly, Arif et al. [64]. also reported significantly lower prevalence of brucellosis in cattle and buffalo farms with owners having university/technical education as compared to those having primary/secondary education. It was also reported that farmers with poor educational staus had no/limited knowledge about brucellosis as compared to educated ones. It might be speculated as a possible reason behind higher prevalence of brucellosis in animals of farmers who had no awareness about brucellosis. Accordingly, farmers with poor educational status are more likely to get infected with brucellosis as an occupational hazard. Al-Shamahy et al. [17]. also reported that humans diagnosed for brucellosis in Yemen had lower level of education as compared to control. It warrants the need to run awareness campaigns about awareness of brucellosis to avoid its animal to animal and zoonotic transmission.

Limitation of our study included the lack of a well-defined data frame pertaining to the number of livestock holdings and rearing of cattle/buffaloes in a scattered manner in both organized and smallholdings with a considerable unregistered population in the study area which might have resulted in selection bias. To mitigate this limitation, we collaborated with the Livestock and Dairy Development Department of the Government of Punjab to include animals from both organized farms and small holdings throughout the study area.

## Conclusions and recommendations

In conclusion, bovine brucellosis is endemic in large ruminants of district Multan of southern Punjab, Pakistan. Considering the comparable preliminary screening efficiencies, the locally manufactured RBPT antigen is advantageous to imported RBPT reagents for being cheaper and thus more economical screening tool. Molecular analysis revealed endemicity of both *B. abortus* and *B. melitensis* in the study area. The herd size, breed, reproductive disorders, mode of insemination, educational status of farmers and lack of awareness of farmers about the disease had significant association with bovine brucellosis in the study area. It is recommended to devise and implement effective brucellosis control strategies comprising (i) active disease surveillance, (ii) awareness campaigns through print and electronic media, (iii) quality assurance of semen to be used for artificial insemination, (iv) capacity building of diagnostic labs for accurate and timely diagnosis of brucellosis and (v) routine vaccination against *Brucella* in endemic regions. Further, the current situation also warrants the need to strengthen interdisciplinary coordination among livestock and human health professionals in one health perspectives to ensure and strengthen the human and animal health care systems in the society.

## Methods

### Study area and target population

**Fig. 1:**
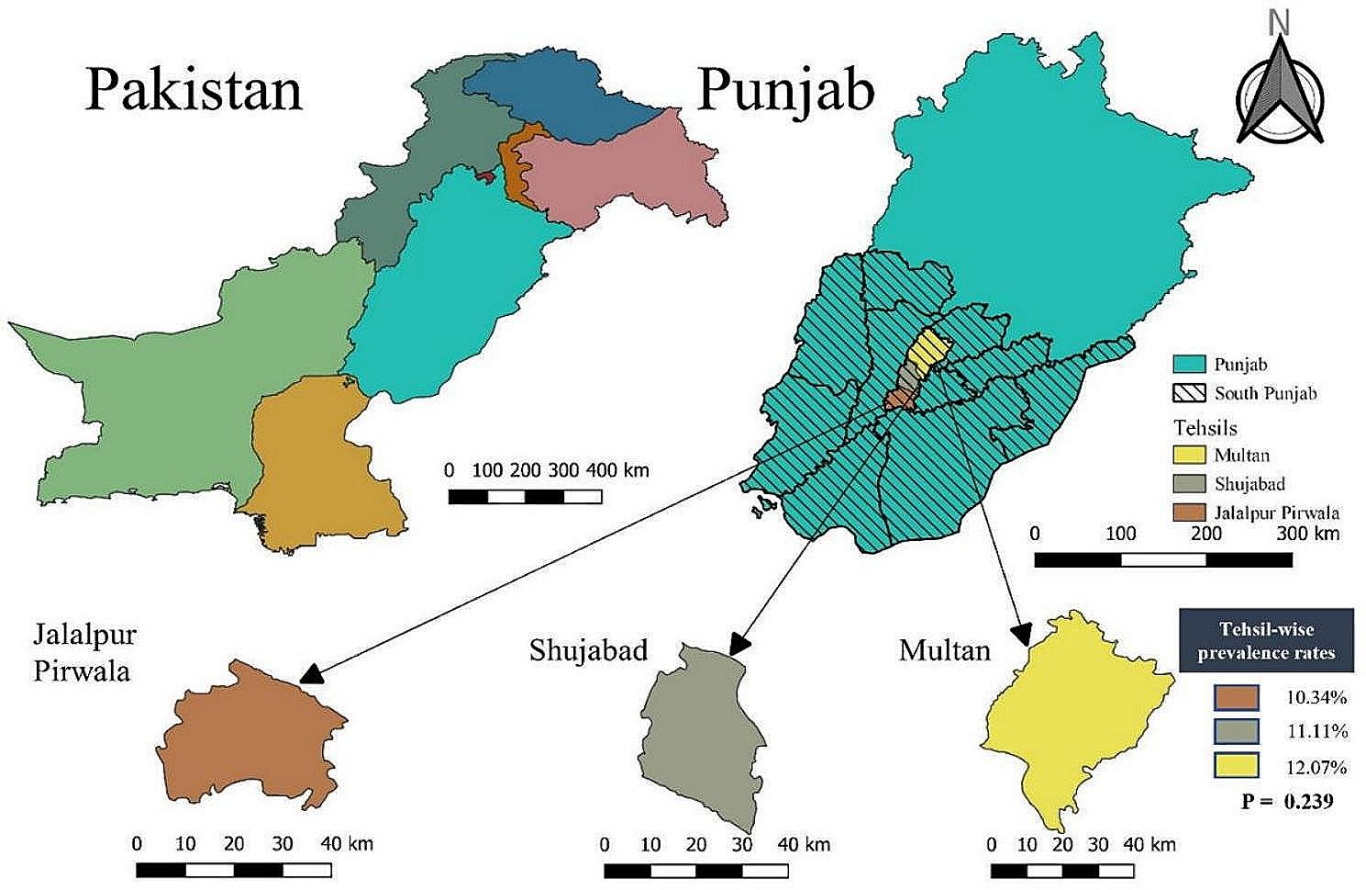
The map of study area showing different Tehsils of district Multan-Pakistan. The study map was developed using QGIS software (version 3.24.2)

This study was conducted from October 2020 to September 2021 in large ruminants (cow and buffalo) located in peri-urban and rural areas of Multan district of Pakistan, located at the bank of river Chenab (Fig. 1). It lies between 30°11′44″N and 71°28′31″E at an elevation of 215 m (740 ft.) above sea-level. It is one of the biggest districts of the southern Punjab, Pakistan with an area of 3,721 km². The study area has extreme climatic conditions. In summer, its temperature rises to 50 °C (maximum) and in winter it lowers down up to 1 °C (minimum). The average precipitation is 127 mm. (http://www.mda.gop.pk/aboutmultan_menu.php; assessed on July 19, 2020).

### Study Design and Sample size

Stratified sampling technique was used to collect the samples from the study area. The target population was divided into two strata viz. cattle and buffaloes and sample size from each stratum was calculated by using the formula [65] as follows:

$$n=\frac{{Z}^{2}\times P\left(1-P\right)}{{\epsilon }^{2}}$$

Where, n= No. of samples; Z= 1.96 (95% level of confidence); $\epsilon$ = level of precision (5%) and P = Expected prevalence (fixed at 20%)

By using the above formula, sample size of each stratum was calculated as 245. Accordingly, a total of 490 blood samples were collected from both cattle and buffalo population of study area.

### Blood sampling

For blood sampling, livestock framers located in peri-urban and rural areas of district Multan were approached with the help of Livestock and Dairy Development Department, Multan Division, Government of the Punjab. After proper restraining, blood sample (5 mL) was aseptically collected from jugular vein of each animal. Out of total, 3 mL blood was shifted to pre-labelled gel & clot activator tubes (BIO-VAC, India) to separate the sera samples and 2 mL was added in Ethylene diamine tetra-acetic acid (EDTA) coated tubes (Xinle Medical, China) to extract the DNA from seropositive animals. All the samples were transferred to One Health Research Lab., Department of Pathobiology, Faculty of Veterinary Sciences, Bahauddin Zakariya University, Multan-Pakistan where sera ware separated in pre-labelled serum vacutainers and stored at -40 °C and blood samples were stored at 4 °C till further use.

### Descriptive epidemiological data collection

The descriptive epidemiological data regarding the pre-disposing factors including (age, breed, gender, body weight, abortion history, repeat breeding, retained fetal membranes, physiological status, educational status of livestock farmers, herd size, puberty, mode of insemination etc.) were collected using a well-designed questionnaire.

### Screening of brucellosis using Rose Bengal plate test (RBPT)

All the collected sera samples were subjected to preliminary screening by RBPT using three different commercially available RBPT reagents viz. i). RBPT antigen, Veterinary Research Institute (VRI), Lahore, Pakistan, ii). Rose Bengal (Rapid slide agglutination antigen), (ID.vet®, France; Cat. # RSA-RB) and iii). Pourquier Rose Bengal Ag (IDEXX, USA; Cat. # P00215). All the tests were performed according to the instructions of respective manufacturers of RBPT reagents. Briefly, for each RBPT reagent, the *Brucella* antigen (30µL) was mixed with an equal volume of serum followed by shaking for 2–4 min. The sera which showed formation of clear agglutination zone (approx. 2 cm diameter) were considered positive and those with no agglutination were considered negative. The known positive and negative controls were also used with each testing session/batch as a reference.

### Serological detection of brucellosis by using Indirect multi-species ELISA Kit

All the collected samples were analyzed using commercially available ELISA kit (ID-Screen® Brucellosis Serum Indirect Multispecies; product ref # BRUS-MS-10P; ID. Vet®, France) to determine the seroprevalence of brucellosis in target population. The ELISA kit has 100% sensitivity and 99.74% specificity as reported by the manufacturer. The test was performed as per the manufacturer’s instructions. The plates were examined using ELISA plate reader (ELx800, BioTek, USA) at 450 nm and data was interpreted using ID. Soft Ver 5.11.6 data analysis software (ID. Vet, France).

### Molecular detection of *Brucella* species by polymerase chain reaction (PCR)

**Fig. 2:**
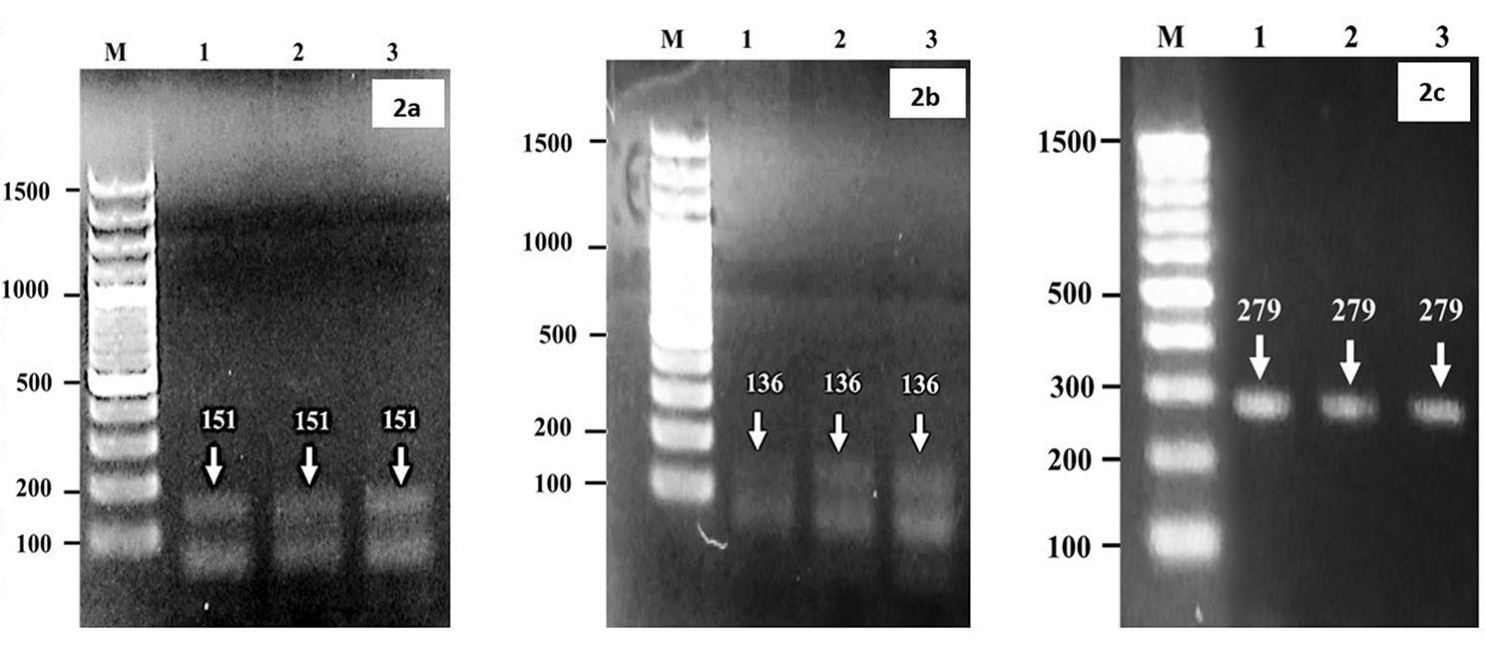
**a-c** Gel Image showing **a**) bands of *Brucella* genus (151 bp), **b**) *B. abortus* (136) and **c**) *B. melitensis* (279) in PCR product by agarose gel electrophoresis

The genomic DNA was extracted from seropositive samples by using commercially available GeneJET-Genomic DNA-Purification Kit (ThermoFisher scientific; Catalog # K0721) as per instructions of manufacturer. All the ELISA positive samples were subjected to extraction and amplification of bacterial genome for the detection of *Brucella* (*B*.) species viz. *B. abortus* and *B. melitensis* by using genus and species-specific primers. Specific primers for *Brucella* Genus with forward primer (5’ to 3’) GCTCGGTTGCCAATATCAATGC and reverse primer (3’ to 5’) GGGTAAAGCGTCGCCAGAAG with product size of 151 bp; for *B. abortus* forward primer GCGGCTTTTCTATCACGGTATTC and reverse primer CATGCGCTATGATCTGGTTACG with product size of 136 bp and for *B. melitensis* forward primer AACAAGCGGCACCCCTAAAA and reverse primer CATGCGCTATGATCTGGTTACG with product size of 279pb were used for amplification [66]. The bands of amplicons as seen by UV-transilluminator (Fig. 2a-c). For PCR reaction, a total of 50 µL reaction mixture containing DreamTaq PCR-Master Mix (2X; 25 µL) (Thermo Scientific, Lithuania), 2 µL of (10 pM) of each primer, 5 µL of extracted DNA and 16 µL of DNase free de-ionized water was prepared. The negative control was a reaction mixture without DNA. Amplification and real-time fluorescence detection was done using a Thermal cycler (MultiGene Optimax, Labnet International, USA). The reaction conditions were as follows: initial denaturation for 10 min at 94 °C, followed by 35 cycles of 94 °C for 1 min, 57 °C (for *Brucella* specie) and 58 °C (for *B. Abortus* and *B. melitensis*) for 30 s, and 72 ° C for 1 min and final extension/elongation for 5 min at 72 °C. The amplified PCR product was subjected to gel electrophoresis using 1.8% agarose gel containing ethidium bromide as a staining reagent followed by visualization of required bands using UV Trans-illuminator (MS Major Science, USA).

### Statistical analysis

Data collected from the questionnaires and laboratory analyses were entered into Microsoft excel spreadsheet (Microsoft Office 365) and association of brucellosis positivity was assessed with independent variables including species, location, age, gender, herd size, breed, parity, pregnancy and puberty status, mode of insemination, farming system, educational status of farmer, farmers’ awareness regarding brucellosis, history of abortion, retention of fetal membranes, and repeat breeding. These associations were analyzed statistically by employing different tests viz. Pearson Chi-square, Odds Ratio and Confidence intervals (95%) using Minitab (v.19) and R (version 4.2.0) with R Studio (version 2022.02.3 + 492) as interface. The differences between variables were considered significant at *P* < 0.05.

## Data Availability

The data that support the findings of this study is available on reasonable request from the corresponding author.

## References

[1] Ullah Q, Jamil T, Melzer F, Saqib M, Hussain MH, Aslam MA et al. Epidemiology and Associated Risk factors for brucellosis in small ruminants kept at Institutional Livestock Farms in Punjab, Pakistan. Front Veterinary Sci. 2020;7(526). 10.3389/fvets.2020.00526 PMC7493811 33117846

[2] Dahourou LD, Ouoba LB, Minoungou L-BG, Tapsoba ARS, Savadogo M, Yougbaré B (2023). Prevalence and factors associated with brucellosis and tuberculosis in cattle from extensive husbandry systems in Sahel and Hauts-Bassins regions, Burkina Faso. Sci Afr.

[3] González-Espinoza G, Arce-Gorvel V, Mémet S, Gorvel J-P (2021). Brucella: reservoirs and niches in animals and humans. Pathogens.

[4] Corbel MJ. Brucellosis: epidemiology and prevalence worldwide. Brucellosis: clinical and laboratory aspects. CRC Press; 2020. pp. 25–40.

[5] Dean AS, Crump L, Greter H, Hattendorf J, Schelling E, Zinsstag J (2012). Clinical manifestations of human brucellosis: a systematic review and meta-analysis. PLoS Negl Trop Dis.

[6] Ibrahim M, Schelling E, Zinsstag J, Hattendorf J, Andargie E, Tschopp R (2021). Sero-prevalence of brucellosis, Q-fever and Rift Valley fever in humans and livestock in Somali Region, Ethiopia. PLoS Negl Trop Dis.

[7] Edao BM, Ameni G, Assefa Z, Berg S, Whatmore AM, Wood JL (2020). Brucellosis in ruminants and pastoralists in Borena, Southern Ethiopia. PLoS Negl Trop Dis.

[8] Holt HR, Bedi JS, Kaur P, Mangtani P, Sharma NS, Gill JPS (2021). Epidemiology of brucellosis in cattle and dairy farmers of rural Ludhiana, Punjab. PLoS Negl Trop Dis.

[9] Jamil T, Kasi KK, Melzer F, Saqib M, Ullah Q, Khan MR (2020). Revisiting brucellosis in small ruminants of western Border areas in Pakistan. Pathogens.

[10] Nejad RB, Krecek RC, Khalaf OH, Hailat N, Arenas Gamboa AM (2020). Brucellosis in the Middle East: current situation and a pathway forward. PLoS Negl Trop Dis.

[11] Dadar M, Tiwari R, Sharun K, Dhama K (2021). Importance of brucellosis control programs of livestock on the improvement of one health. Vet Q.

[12] Zeng J, Duoji C, Yuan Z, Yuzhen S, Fan W, Tian L (2017). Seroprevalence and risk factors for bovine brucellosis in domestic yaks (Bos grunniens) in Tibet, China. Trop Anim Health Prod.

[13] Joseph OA, Oluwatoyin AV, Comfort AM, Judy S, Babalola CSI (2015). Risk factors associated with brucellosis among slaughtered cattle: epidemiological insight from two metropolitan abattoirs in Southwestern Nigeria. Asian Pac J Trop Dis.

[14] Makita K, Fèvre EM, Waiswa C, Eisler MC, Thrusfield M, Welburn SC (2011). Herd prevalence of bovine brucellosis and analysis of risk factors in cattle in urban and peri-urban areas of the Kampala economic zone, Uganda. BMC Vet Res.

[15] Aune K, Rhyan J, Russell R, Roffe T, Corso B (2012). Environmental persistence of Brucella abortus in the Greater Yellowstone Area. J Wildl Manag.

[16] Hegazy YM, Moawad A, Osman S, Ridler A, Guitian J (2011). Ruminant brucellosis in the Kafr El Sheikh Governorate of the Nile Delta, Egypt: prevalence of a neglected zoonosis. PLoS Negl Trop Dis.

[17] Al-Shamahy H, Whitty C, Wright S (2000). Risk factors for human brucellosis in Yemen: a case control study. Epidemiol Infect.

[18] Briones G, Iñón de Iannino N, Roset M, Vigliocco A, Paulo PS, Ugalde RA (2001). Brucella abortus cyclic beta-1,2-glucan mutants have reduced virulence in mice and are defective in intracellular replication in HeLa cells. Infect Immun.

[19] Memish ZA, Balkhy HH (2004). Brucellosis and international travel. J Travel Med.

[20] Seleem MN, Boyle SM, Sriranganathan N (2010). Brucellosis: a re-emerging zoonosis. Vet Microbiol.

[21] Zhang N, Huang D, Wu W, Liu J, Liang F, Zhou B (2018). Animal brucellosis control or eradication programs worldwide: a systematic review of experiences and lessons learned. Prev Vet Med.

[22] Iqbal M, Fatmi Z, Khan MA (2020). Brucellosis in Pakistan: a neglected zoonotic disease. JPMA The Journal of the Pakistan Medical Association.

[23] Franc KA, Krecek RC, Häsler BN, Arenas-Gamboa AM (2018). Brucellosis remains a neglected disease in the developing world: a call for interdisciplinary action. BMC Public Health.

[24] Cadmus S, Adesokan H, Stack J (2008). The use of the milk ring test and rose bengal test in brucellosis control and eradication in Nigeria. J S Afr Vet Assoc.

[25] Roushan MR, Amiri MJ, Laly A, Mostafazadeh A, Bijani A (2010). Follow-up standard agglutination and 2-mercaptoethanol tests in 175 clinically cured cases of human brucellosis. Int J Infect Diseases: IJID : Official Publication Int Soc Infect Dis.

[26] Jamil T, Melzer F, Saqib M, Shahzad A, Khan Kasi K, Hammad Hussain M (2020). Serological and Molecular Detection of Bovine Brucellosis at Institutional Livestock Farms in Punjab, Pakistan. Int J Environ Res Public Health.

[27] Khan AU, Melzer F, Hendam A, Sayour AE, Khan I, Elschner MC et al. Seroprevalence and Molecular Identification of Brucella spp. in bovines in Pakistan—investigating Association with risk factors using machine learning. Front Vet Sci. 2020;7. 10.3389/fvets.2020.594498 PMC7738322 33344532

[28] Baig S. Seroprevalence of bovine brucellosis and analysis of risk factors in cattle and livestock Handler’s in Gilgit – Pakistan, 2019. Int J Infect Dis. 2020;101:534.

[29] Villanueva MA, Mingala CN, Tubalinal GAS, Gaban PBV, Nakajima C, Suzuki Y. Emerging infectious diseases in water buffalo: An economic and public health concern. Emerging Infectious Diseases in Water Buffalo-An Economic and Public Health Concern. 2018.

[30] Pudake R, Jain U, Kole C, Shakya S, Saxena K. Nano-Biosensing Devices Detecting Biomarkers of Communicable and Non-communicable Diseases of Animals. Biosensors in Agriculture: Recent Trends and Future Perspectives. 2020:415 – 34.

[31] Deresa B, Tulu D, Deressa FB (2020). Epidemiological Investigation of Cattle Abortion and Its Association with brucellosis in Jimma Zone, Ethiopia. Veterinary Medicine: Research and Reports.

[32] Ukwueze KO, Ishola OO, Dairo MD, Awosanya EJ, Cadmus SI (2020). Seroprevalence of brucellosis and associated factors among livestock slaughtered in Oko-Oba abattoir, Lagos State, southwestern Nigeria. Pan Afr Med J.

[33] Ntivuguruzwa JB, Kolo FB, Gashururu RS, Umurerwa L, Byaruhanga C, van Heerden H (2020). Seroprevalence and Associated Risk factors of bovine brucellosis at the Wildlife-Livestock-Human interface in Rwanda. Microorganisms.

[34] Fero E, Juma A, Koni A, Boci J, Kirandjiski T, Connor R (2020). The seroprevalence of brucellosis and molecular characterization of Brucella species circulating in the beef cattle herds in Albania. PLoS ONE.

[35] Shrimali M, Patel S, Chauhan H, Chandel B, Patel A, Sharma K (2019). Seroprevalence of brucellosis in bovine. Int J Curr Microbiol App Sci.

[36] Madut NA, Muwonge A, Nasinyama GW, Muma JB, Godfroid J, Jubara AS (2018). The sero-prevalence of brucellosis in cattle and their herders in Bahr El Ghazal region, South Sudan. PLoS Negl Trop Dis.

[37] Pathak AD, Dubal ZB, Karunakaran M, Doijad SP, Raorane AV, Dhuri RB et al. Apparent seroprevalence, isolation and identification of risk factors for brucellosis among dairy cattle in Goa, India. Comparative immunology, microbiology and infectious diseases. 2016;47:1–6. 10.1016/j.cimid.2016.05.004 27477501

[38] Asgedom H, Damena D, Duguma R (2016). Seroprevalence of bovine brucellosis and associated risk factors in and around Alage district. Ethiopia SpringerPlus.

[39] Awah-Ndukum J, Mouiche MMM, Kouonmo-Ngnoyum L, Bayang HN, Manchang TK, Poueme RSN (2018). Seroprevalence and risk factors of brucellosis among slaughtered indigenous cattle, abattoir personnel and pregnant women in Ngaoundéré, Cameroon. BMC Infect Dis.

[40] Chaka H, Aboset G, Garoma A, Gumi B, Thys E (2018). Cross-sectional survey of brucellosis and associated risk factors in the livestock-wildlife interface area of Nechisar National Park, Ethiopia. Trop Anim Health Prod.

[41] Bifo H, Gugsa G, Kifleyohannes T, Abebe E, Ahmed M (2020). Sero-prevalence and associated risk factors of bovine brucellosis in Sendafa, Oromia Special Zone surrounding Addis Ababa, Ethiopia. PLoS ONE.

[42] Kamga RMN, Silatsa BA, Farikou O, Kuiate JR, Simo G (2020). Detection of Brucella antibodies in domestic animals of southern Cameroon: implications for the control of brucellosis. Vet Med Sci.

[43] Yanti Y, Sumiarto B, Kusumastuti T, Panus A, Sodirun S, editors. Seroprevalence and risk factors of brucellosis and the brucellosis model at the individual level of dairy cattle in the West Bandung District, Indonesia, Veterinary World, 14 (1): 1–102021: Abstract. 10.14202/vetworld.2021.1-10 PMC7896884 33642780

[44] Boukary AR, Saegerman C, Abatih E, Fretin D, Alambédji Bada R, De Deken R (2013). Seroprevalence and potential risk factors for Brucella Spp. Infection in traditional cattle, Sheep and Goats Reared in Urban, Periurban and Rural areas of Niger. PLoS ONE.

[45] Ali S, Akhter S, Neubauer H, Melzer F, Khan I, Abatih EN (2017). Seroprevalence and risk factors associated with bovine brucellosis in the Potohar Plateau, Pakistan. BMC Res Notes.

[46] Khan MR, Rehman A, Khalid S, Ahmad MUD, Avais M, Sarwar M (2021). Seroprevalence and Associated Risk factors of bovine brucellosis in District Gujranwala, Punjab, Pakistan. Animals.

[47] Rodriguez-Morales J. A. Climate change, climate variability and brucellosis. Recent patents on anti-infective drug discovery. 2013;8(1):4–12. 10.2174/1574891x11308010003 22873353

[48] Khan I, Ali S, Hussain R, Raza A, Younus M, Khan N (2021). Serosurvey and potential risk factors of brucellosis in dairy cattle in peri-urban production system in Punjab, Pakistan. Pak Vet J.

[49] Bakhtullah FP, Shahid M, Basit A, Khan MA, Gul S, Wazir I (2014). Sero–prevalence of brucellosis in cattle in southern area of Khyber Pakhtunkhwa, Pakistan. Res J Vet Pract.

[50] Ahmad T, Khan I, Razzaq S, Akhtar R. Prevalence of bovine brucellosis in Islamabad and rawalpindi districts of Pakistan. Pakistan J Zool. 2017;49(3).

[51] Sima DM, Ifa DA, Merga AL, Tola EH (2021). Seroprevalence of bovine brucellosis and Associated Risk factors in Western Ethiopia. Veterinary Medicine: Research and Reports.

[52] Haileselassie M, Kalayou S, Kyule M, Asfaha M, Belihu K. Effect of Brucella infection on reproduction conditions of female breeding cattle and its public health significance in Western Tigray, northern Ethiopia. Veterinary medicine international. 2011;2011. 10.4061/2011/354943 PMC3142704 21822466

[53] Saeed U, Ali S, Latif T, Rizwan M, Iftikhar A, Ghulam Mohayud Din Hashmi S (2020). Prevalence and spatial distribution of animal brucellosis in central Punjab, Pakistan. Int J Environ Res Public Health.

[54] Patel M, Patel P, Prajapati M, Kanani A, Tyagi K, Fulsoundar A (2014). Prevalence and risk factor’s analysis of bovine brucellosis in peri-urban areas under intensive system of production in Gujarat, India. Vet World.

[55] França T, Ishikawa L, Zorzella-Pezavento S, Chiuso-Minicucci F, da Cunha MdLRdS, Sartori A (2009). Impact of malnutrition on immunity and infection. J Venom Anim Toxins Including Trop Dis.

[56] Rahman M, Faruk M, Her M, Kim J, Kang S, Jung S (2011). Prevalence of brucellosis in ruminants in Bangladesh. Vet Med.

[57] Deka RP, Shome R, Dohoo I, Magnusson U, Randolph DG, Lindahl JF (2021). Seroprevalence and risk factors of Brucella infection in dairy animals in urban and rural areas of Bihar and Assam, India. Microorganisms.

[58] Aulakh H, Patil P, Sharma S, Kumar H, Mahajan V, Sandhu K (2008). A study on the epidemiology of bovine brucellosis in Punjab (India) using milk-ELISA. Acta Vet Brno.

[59] Khan AU, Sayour AE, Melzer F, El-Soally SAGE, Elschner MC, Shell WS (2020). Seroprevalence and molecular identification of Brucella spp. in camels in Egypt. Microorganisms.

[60] Dinka H, Chala R (2009). Seroprevalence study of bovine brucellosis in pastoral and agro-pastoral areas of East Showa Zone, Oromia Regional State, Ethiopia. American-Eurasian J Agricultural Environ Sci.

[61] Garry F. Chapter 15 - Miscellaneous Infectious Diseases. In: Divers TJ, Peek SF, editors. Rebhun’s Diseases of Dairy Cattle (Second Edition). Saint Louis: W.B. Saunders; 2008. p. 606 – 39.

[62] Batista HR, Passos CTS, Nunes Neto OG, Sarturi C, Coelho APL, Moreira TR (2020). Factors associated with the prevalence of antibodies against Brucella abortus in water buffaloes from Santarém, Lower Amazon region, Brazil. Transbound Emerg Dis.

[63] Cárdenas L, Peña M, Melo O, Casal J (2019). Risk factors for new bovine brucellosis infections in Colombian herds. BMC Vet Res.

[64] Arif S, Thomson PC, Hernandez-Jover M, McGill DM, Warriach HM, Hayat K (2019). Bovine brucellosis in Pakistan; an analysis of engagement with risk factors in smallholder farmer settings. Veterinary Med Sci.

[65] Pourhoseingholi MA, Vahedi M, Rahimzadeh M (2013). Sample size calculation in medical studies. Gastroenterol Hepatol Bed Bench.

[66] Probert WS, Schrader KN, Khuong NY, Bystrom SL, Graves MH (2004). Real-time multiplex PCR assay for detection of < i > Brucella spp., <i > B. Abortus, and < i > B. melitensis</i >. J Clin Microbiol.

